# Hippo transducer TAZ promotes epithelial mesenchymal transition and supports pancreatic cancer progression

**DOI:** 10.18632/oncotarget.5772

**Published:** 2015-09-21

**Authors:** Dacheng Xie, Jiujie Cui, Tian Xia, Zhiliang Jia, Liang Wang, Wenfei Wei, Anna Zhu, Yong Gao, Keping Xie, Ming Quan

**Affiliations:** ^1^ Department of Oncology, Shanghai Tongji University Affiliated East Hospital, Shanghai, People's Republic of China; ^2^ Department of Gastroenterology, Hepatology and Nutrition, The University of Texas MD Anderson Cancer Center, Houston, TX, USA; ^3^ Department of Gastroenterology, Shanghai Changhai Hospital, Second Military Medical University, Shanghai, People's Republic of China

**Keywords:** TAZ, EMT, proliferation, metastasis, pancreatic cancer

## Abstract

Transcriptional co-activator with PDZ binding motif (TAZ) is a transducer of the Hippo pathway and promotes cancer development and progression. In the present study, we sought to determine the roles and underlying mechanisms of elevated expression and activation of TAZ in pancreatic cancer development and progression. The mechanistic role of TAZ and Hippo signaling in promotion of pancreatic cancer development and progression was examined using cell culture, molecular biology, and mouse models. The relevance of our experimental and mechanistic findings was validated using human pancreatic tumor specimens. We found that TAZ expression was markedly higher in pancreatic tumors than in normal pancreatic tissue. Further analysis of the correlation of TAZ expression with tissue microarray clinicopathologic parameters revealed that this expression was positively associated with tumor differentiation. Also, TAZ expression was higher in pancreatic cancer cell lines than in pancreatic ductal epithelial cells. TAZ activation in pancreatic cancer cells promoted their proliferation, migration, invasion, and epithelial-mesenchymal transition. Further mechanistic studies demonstrated that aberrant expression and activation of TAZ in pancreatic cancer cells resulted from suppression of the expression of Merlin, a positive regulator upstream of the Hippo pathway, and that the oncogenic function of TAZ in pancreatic cancer cells was mediated by TEA/ATTS domain transcription factors. Therefore, TAZ functioned as an oncogene and promoted pancreatic cancer epithelial-mesenchymal transition and progression. TAZ thus may be a target for effective therapeutic strategies for pancreatic cancer.

## INTRODUCTION

Pancreatic cancer, also known as pancreatic ductal adenocarcinoma, is a deadly disease, with a 5-year survival rate less than 6% and incidence that is increasing annually [1, 2]. Although surgery for pancreatic cancer has improved, most cases are diagnosed at a late stage, losing the opportunity for resection. Thus, new targets for early detection and treatment of pancreatic cancer are urgently needed. Recent studies revealed that pancreatic cancer is essentially a genetic disease, resulting from genetic and epigenetic alterations [3-5]. Therefore, identification of key genetic and epigenetic alterations that promote pancreatic cancer development and progression should help the generation of novel strategies for early detection and treatment of pancreatic cancer.

Transcriptional co-activator with PDZ binding motif (TAZ), also known as WW domain-containing transcription regulator-1, is a transducer of the Hippo pathway. This pathway is an essential regulator of organ size, stem cell maintenance, and tumorigenesis [6, 7]. In mammals, with activation of the Hippo pathway, the pathway kinases MST1 and MST2 phosphorylate LATS1 and LATS2 and inactivate TAZ and its paralog Yes-associated protein (YAP) [7]. In contrast, inactivation of the Hippo pathway results in TAZ/YAP nuclear translocation, with interacting with TEA/ATTS domain (TEAD) transcription factors and transactivating their downstream targets, including connective tissue growth factor (CTGF) and survivin, and involving in cell proliferation, stem cell self-renewal, and tumorigenesis [8, 9]. The importance of this pathway in human has been elegantly reviewed [10, 11]. There are genetically engineered mouse strains of TAZ deletion, which show extensive abnormalities, particularly in the kidneys and lungs [12-14]. Authors have reported that TAZ is aberrantly expressed and activated in cases of different cancers, including lung, breast, colorectal, ovarian, brain, liver, and oral cancer, and promotes tumor growth, metastasis, epithelial-mesenchymal transition (EMT), and stem cell maintenance [15-21]. However, the expression and roles of TAZ in pancreatic cancer development and progression have yet to be demonstrated.

Merlin, the protein encoded by the NF2 gene, is a member of the band 4.1 families of cytoskeleton-associated proteins, which link the integral membrane proteins with the actin cytoskeleton [22, 23]. Investigators first identified Merlin as being associated with neurofibromatosis type 2 and that it functions as a tumor suppressor [24]. A number of studies demonstrated that Merlin is a versatile tumor suppressor that can inhibit cancer cell proliferation and motility by modulating a wide range of signaling pathways [25]. Furthermore, mutation of the NF2 gene and loss of Merlin protein occur in many different types of cancer, suggesting a general tumor-suppressive role for Merlin [26-31]. In a study of Ezrin, another member of the band 4.1 protein superfamily, overexpression of Merlin inhibited SW1990 PDA cell proliferation, migration, and adhesion [32]. Earlier study has indicated the crosstalk between YAP/TAZ pathway and Merlin/NF2 signaling [33]. However, their interacting roles and mechanism in PDA development and progression have remained unclear.

In the present study, we sought to investigate the expression of TAZ and determine its roles in pancreatic cancer development and progression. We found that expression of TAZ was markedly increased in both pancreatic tumors and pancreatic cancer cell lines. Overexpression of TAZ increased pancreatic cancer cell proliferation, migration, invasion, and EMT, whereas depletion of TAZ did the opposite. Further studies demonstrated that Merlin regulated the expression and activation of TAZ and that TEADs mediated the oncogenic roles of TAZ regarding pancreatic cancer.

## RESULTS

### Expression of TAZ is correlated clinically with pancreatic cancer development and progression

**Figure 1:**
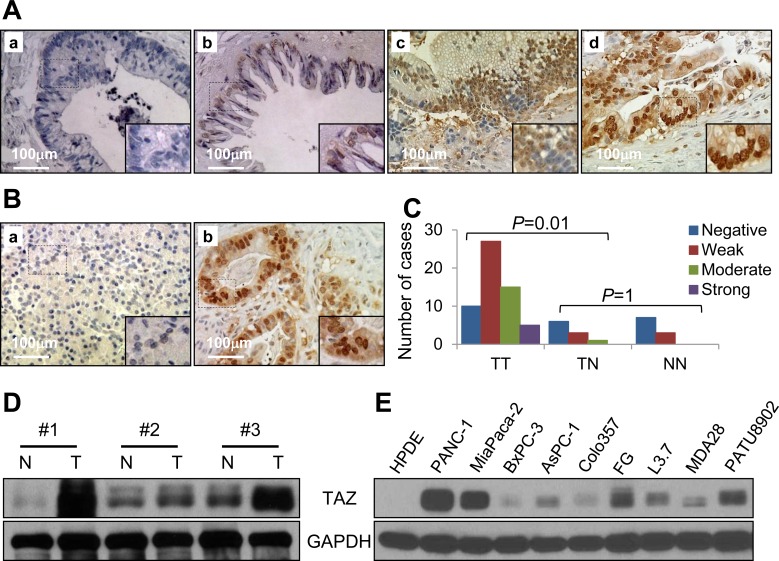
Expression of TAZ in pancreatic tumors and pancreatic cancer cell lines Pancreatic tumor specimens in a TMA were immunostained for TAZ protein using a specific anti-TAZ antibody. **A.** Stains of pancreatic tumor specimens showing **a.** negative TAZ staining, **b.** weakly positive TAZ staining, **c.** moderately positive TAZ staining, and **d.** strongly positive TAZ staining. **B.** Stains of nonmalignant pancreatic tissue specimens showing negative TAZ staining **a.**, while pancreatic cancer tissue showing positive TAZ staining **b.**. **C.** Graph showing that the expression of TAZ was significantly higher in tumor (TT) than in tumor-adjacent normal tissue (TN) specimens and that TAZ expression did not differ significantly between TN and normal tissue (NN) specimens. **D.** Western blot verifying the expression of TAZ protein in paired normal pancreatic tissue (N) and pancreatic tumor (T) specimens. **E.** Western blot showing the expression of TAZ protein in pancreatic cancer cell lines.

To determine the roles of TAZ in pancreatic cancer development and progression, we first analyzed the expression of TAZ in a pancreatic cancer tissue microarray (TMA), which contained 57 primary pancreatic tumor, 10 tumor-adjacent normal pancreatic tissue, and 10 normal pancreatic tissue specimens, using immunohistochemistry. Authors have described the clinicopathologic characteristics of this TMA [34]. The results of this analysis demonstrated that nuclei were positive for TAZ in pancreatic tumor specimens but negative or weakly positive for it in tumor-adjacent normal and normal pancreatic tissue specimens (Figure 1A, 1B). Furthermore, the expression of TAZ in tumor specimens was much higher than that in nonmalignant tissue specimens (Figure 1C). We then investigated the correlation of TAZ expression with clinicopathologic parameters for the TMA. We found that expression of TAZ did not correlate significantly with age, sex, T stage, N stage, or TNM stage in the pancreatic cancer patients from whom the TMA specimens were obtained (*p* > 0.05) but correlated positively with tumor differentiation (*p* = 0.020) (Table 1). Consistently, the expression of TAZ protein in pancreatic tumor specimens was much higher than that in normal tissue specimens according to Western blot analysis (Figure 1D). Also, in a separate experiment, the expression of TAZ protein was higher in human pancreatic cancer cell lines than in immortalized human pancreatic ductal epithelial cells (Figure 1E). These data indicated that TAZ may be an oncogene for pancreatic cancer and and promotes pancreatic cancer development and progression.

**Table 1:** TAZ expression in and clinicopathologic characteristics for the human pancreatic cancer TMA

Characteristic	Cases (*n* = 57)	TAZ protein expression in tumor tissue (*n*)				*p*
		Negative	Weak	Moderate	Strong	
Age, years						
<60	39	8	19	8	4	0.562
≥60	18	2	8	7	1	
Sex						
Male	36	7	15	9	5	0.301
Female	21	3	12	6	0	
T stage						
T1	1	0	1	0	0	0.850
T2	15	2	9	3	1	
T3	40	8	17	11	4	
T4	1	0	0	1	0	
N stage						
N0	52	10	26	12	4	0.134
N1	5	0	1	3	1	
TNM stage						
I	12	2	8	2	0	0.147
II	39	8	18	10	3	
III	5	0	1	3	1	
IV	1	0	0	0	1	
Differentiation						
Well	11	2	8	1	0	0.020*
Moderate	25	3	13	8	1	
Poor	11	2	1	5	3	

* *P* values were based on the Fisher exact test. The total number of cases with differentiation statuses was 47.

### TAZ promotes the growth of pancreatic cancer cells *in vitro* and *in vivo*

**Figure 2:**
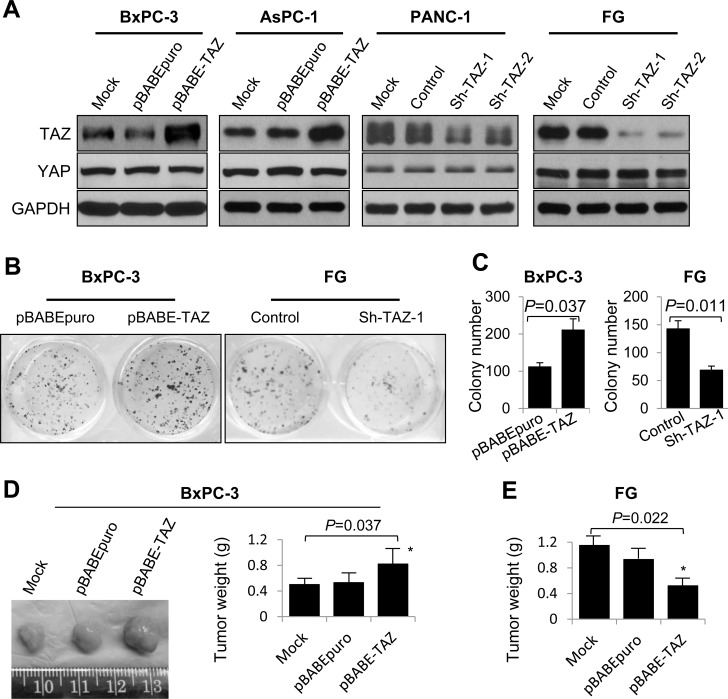
Effect of altered TAZ expression on pancreatic cancer cell growth *in vitro* and *in vivo* **A.** Western blots showing altered expression of TAZ and YAP in pancreatic cancer cell lines with stable overexpression of TAZ (BxPC-3 and AsPC-1) or depletion of TAZ (FG and PANC-1). **B.** and **C.** A colony formation assay was performed using 24-well plates, and the resulting BxPC-3 and FG cell colonies were counted 14 days after the cells were seeded. (**D** and **E**) BxPC-3 cells with TAZ overexpression **D.** and FG cells with TAZ depletion **E.** were injected subcutaneously into the right scapular regions of nude mice (*n* = 5). The resulting tumors were removed from the mice and weighed. The data are presented as the mean ± standard error of the mean from three independent experiments. *P* values are provided, as comparisons were performed as indicated.

To further investigate the effect of TAZ on pancreatic cancer development and progression, we generated BxPC-3 and AsPC-1 cells with stable overexpression of TAZ (BxPC-3/AsPC-1-pBABE-TAZ) and PANC-1 and FG cells with stable depletion of TAZ (PANC-1/FG-Sh-TAZ-1 and -Sh-TAZ-2) (Figure 2A). We performed a colony formation assay to analyze the effect of altered expression of TAZ on pancreatic cancer cell proliferation *in vitro*. As shown in Figure 2B and 2C, elevated TAZ expression significantly increased BxPC-3 cell colony formation, whereas knockdown of TAZ expression suppressed FG cell colony formation. We then determined the effect of altered TAZ expression on pancreatic tumor growth *in vivo*. As shown in Figure 2D and 2E, increased expression of TAZ significantly promoted the growth of tumors induced by BxPC-3 cells, whereas depletion of TAZ markedly suppressed the growth of subcutaneous tumors induced by FG cells. These data demonstrated that TAZ promoted pancreatic cancer cell growth *in vitro* and *in vivo* and supported that TAZ functions as an oncogene in pancreatic cancer cases.

### TAZ promotes the migration, invasion, and EMT of pancreatic cancer cells

**Figure 3:**
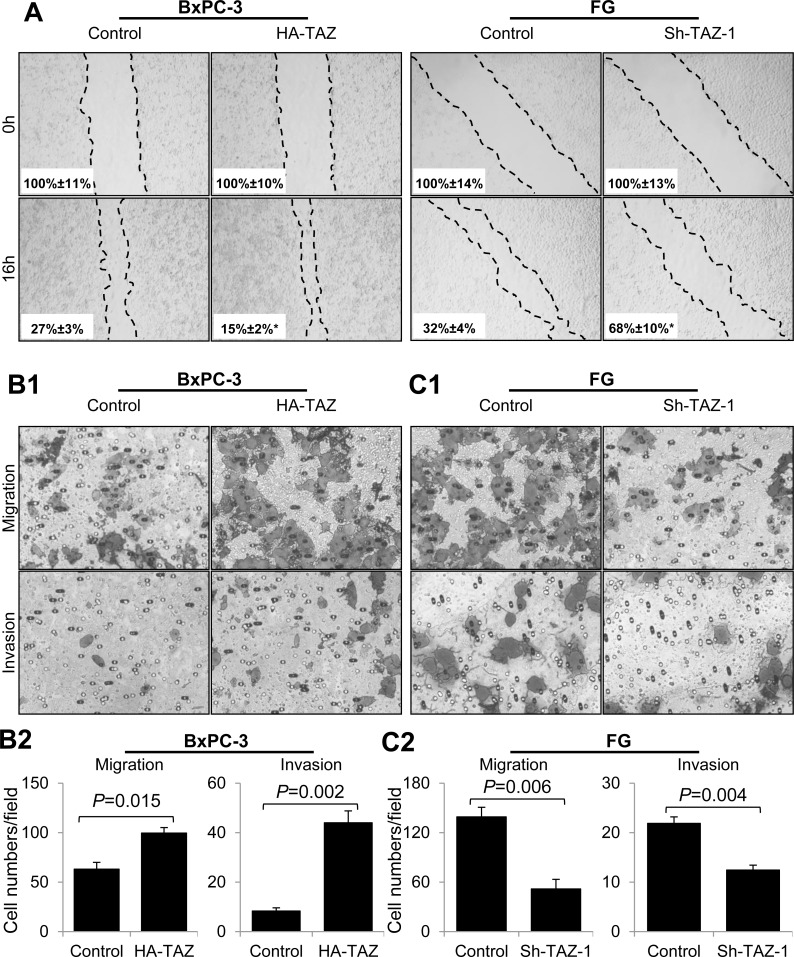
Effect of altered expression of TAZ on pancreatic cancer cell migration and invasion BxPC-3 cells were transiently transfected with HA-TAZ or a control vector, and FG cells were transfected with Sh-TAZ-1 or a control vector. Cell scratch-wound, transwell migration, and invasion assays were used as described in Materials and Methods. Representative photos of wound healing, migration and invasion are shown. The data are presented as the mean ± standard error of the mean from three independent experiments. *P* values are provided, as comparisons were performed as indicated.

Given that TAZ is reported to promote the migration and invasion of neuroblastoma cells, we transfected BxPC-3 cells with hemagglutinin-tagged TAZ (HA-TAZ) or a control vector and FG cells with Sh-TAZ-1 or a control vector and investigated the roles of TAZ in pancreatic cancer migration and invasion [35]. In a scratch-wound assay, elevated TAZ expression increased the flattening and spreading activity of BxPC-3, whereas depletion of TAZ led to suppressed flattening and spreading activity of FG cells (Figure 3A). Similarly, increased expression of TAZ promoted the migration and invasion of BxPC-3 cells (Figure 3B), whereas decreased expression of TAZ attenuated the migration and invasion of FG cells (Figure 3C).

**Figure 4:**
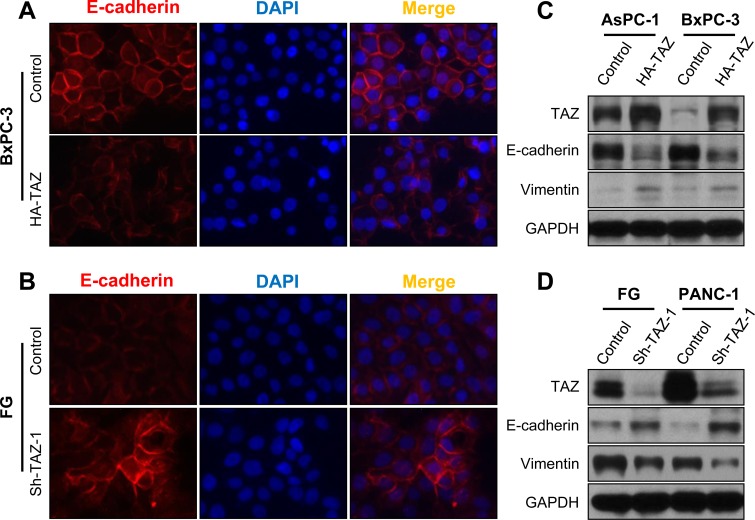
Influence of TAZ expression on the EMT phenotype of pancreatic cancer cells AsPC-1 and BxPC-3 cells were transfected with HA-TAZ or a control vector, and FG and PANC-1 cells were transfected with Sh-TAZ-1 or a control vector. **A.** and **B.** Immunofluorescent stains showing E-cadherin expression in BxPC-3 and FG cells. DAPI, 4′,6-diamidino-2-phenylindole. **C.** and **D.** Western blots showing an abundance of the EMT markers E-cadherin and vimentin in pancreatic cancer cells.

Moreover, EMT is the basis for high metastatic potential of cancer cells, and authors have reported that TAZ expression promotes the EMT of many types of cancer cells [21, 36, 37]. We therefore determined the effect of altered expression of TAZ on the epithelial and/or mesenchymal phenotype of pancreatic cancer cells. As shown in Figure 4A and 4B, in pancreatic cancer cells, overexpression of TAZ markedly decreased the expression of E-cadherin but increased the expression of vimentin. In contrast, depletion of TAZ resulted in increased expression of E-cadherin but decreased expression of vimentin (Figure 4C, 4D). These data indicated that TAZ affected the epithelial and mesenchymal phenotype of pancreatic cancer cells.

### Merlin regulates the expression, nuclear localization, and transcriptional activity of TAZ via the Hippo pathway

**Figure 5:**
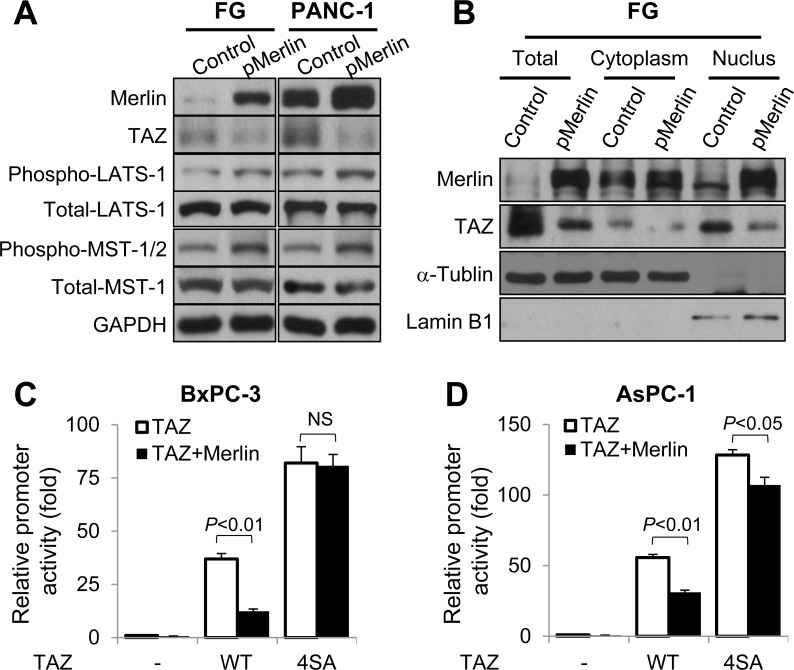
Regulation of the expression, nuclear localization, and transcriptional activity of TAZ by Merlin *via* the Hippo pathway **A.** FG and PANC-1 cells were transfected with a Merlin overexpression vector or control vector. The Western blot shows the expression of Merlin, TAZ, LATS1, phosphorylated LATS1 (phospho-LATS1), MST1, phosphorylated MST1/2 (phospho-MST1/2), and the internal control GAPDH in the cells. **B.** FG cells were transfected with a Merlin overexpression vector or control vector for 48 h, and cytosolic and nuclear proteins were extracted. The Western blot shows the levels of TAZ expression in the cells. (C and D) The 8×GTIIC-luciferase reporter was co-transfected into BxPC-3 **C.** and AsPC-1 **D.** cells with a control vector alone or with pMerlin, HA-TAZ alone or with pMerlin, or 4SA alone or pMerlin for 24 h. The graphs show the resulting promoter activity in the cells as analyzed using a dual luciferase assay.

The data described above clearly demonstrated that TAZ promoted pancreatic cancer development and progression. However, the mechanism of elevated expression and activation of TAZ in pancreatic cancer has yet to be studied. Merlin is reported to function upstream of the Hippo pathway. Specifically, by activating MST1/2 and LATS1/2, Merlin suppresses YAP and TAZ expression and activity and cell proliferation, survival, and motility [38]. Furthermore, authors reported that expression of Merlin was decreased in pancreatic cancer [32]. Thus, we further investigated the regulatory effect of Merlin on TAZ expression in pancreatic cancer cells. We transfected FG and PANC-1 cells with a Merlin expression vector and control vector and found that restored expression of Merlin did not significantly affect the levels of LATS1 or MST1 expression but led to increased LATS1 and MST1/2 phosphorylation and decreased TAZ expression (Figure 5A). In addition, researchers demonstrated that activation of the Hippo pathway results in decreased nuclear localization of TAZ [7]. Therefore, we then investigated the effect of Merlin on this nuclear localization. We transfected FG cells with a Merlin expression vector or control vector and analyzed the nuclear localization of TAZ using Western blotting. As shown in Figure 5B, restored expression of Merlin decreased the expression of TAZ protein in the nuclei.

The MST and LATS kinases in the Hippo pathway phosphorylate TAZ in four HXRXXS motifs, and four serine residues (S66, S89, S177, and S311) are located in the HXRXXS motifs. Replacement of these residues with alanine produces unphosphorylated TAZ (termed 4SA), which is localized in nuclei and constitutively activated [19, 37]. To further investigate the roles of the Hippo pathway in mediation of the suppressive function of Merlin regarding TAZ expression, we analyzed the regulatory effect of Merlin on TAZ's transcriptional activity. We found that co-transfection of BxPC-3 and AsPC-1 cells with Merlin and TAZ markedly attenuated the transcriptional activity of 8×GTIIC-luciferase, a YAP/TAZ-responsive reporter, whereas co-transfection with Merlin and 4SA had a limited effect on this activity of 8×GTIIC-luciferase (Figure 5C, 5D). These data demonstrated that Merlin negatively regulated the expression and activation of TAZ via the Hippo pathway in pancreatic cancer cells.

### TEAD transcription factors mediate the oncogenic function of TAZ in pancreatic cancer cells

**Figure 6:**
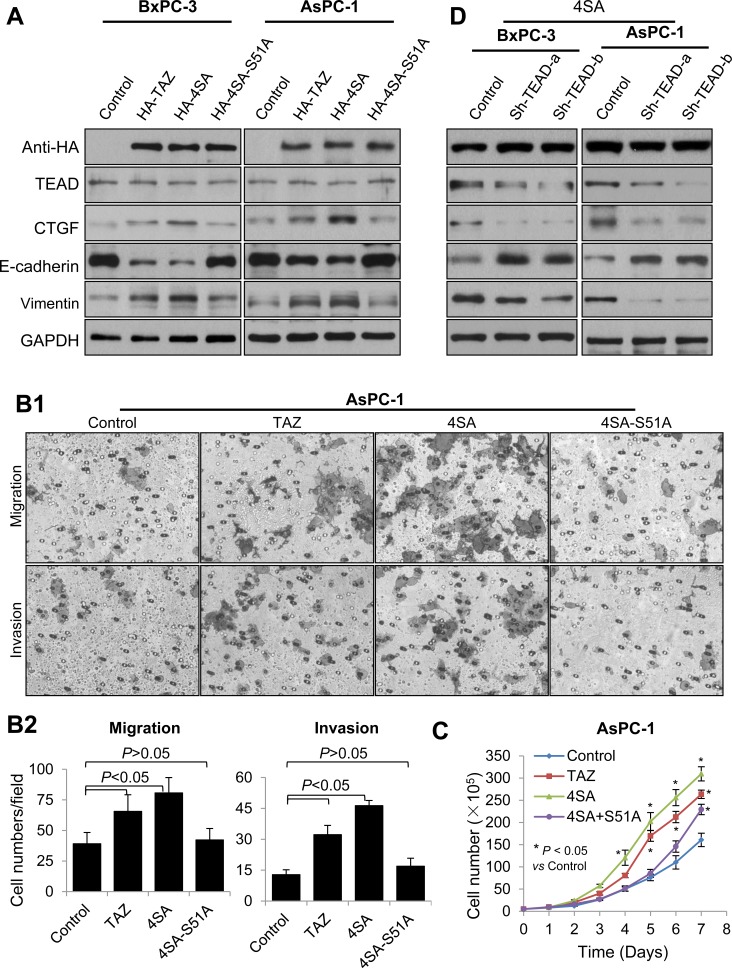
Mediation of the oncogenic function of TAZ in pancreatic cancer cells by TEAD transcription factors **A.** BxPC-3 and AsPC-1 cells were transfected with HA-TAZ, HA-4SA, HA-4SA-S51A, or a control vector. The Western blots show the expression of TAZ and its mutants, TEADs, CTGF, E-cadherin, and vimentin in the cells. **B.** AsPC-1 cells were transfected with a control vector, HA-TAZ, HA-4SA, or HA-4SA-S51A, and transwell migration and invasion assays were used as described in Materials and Methods. Representative photographs of the cell migration and invasion are shown. **C.** Graph showing the AsPC-1 cell growth as assessed via cell counting at the indicated time points. The data are presented as the mean ± standard error of the mean from three independent experiments. **D.** HA-4SA was co-transfected into BxPC-3 and AsPC-1 cells with control vector or shRNAs of TEAD. The Western blots show the expression of TAZ mutants, TEADs, CTGF, E-cadherin and vimentin in the cells.

TAZ interacts with a series of transcription factors, but TEAD transcription factors play prominent roles in TAZ-mediated cell growth and EMT. To test this in pancreatic cancer cases, we first transfected BxPC-3 and AsPC-1 cells with a control vector, TAZ, 4SA, or 4SA-S51A, which had an additional point mutation of 4SA in the TEAD-binding domain and lacked TEAD binding [19, 36]. Western blot analysis demonstrated that TAZ and 4SA increased the expression of CTGF, a typical target gene of TEADs, and that 4SA was more effective than TAZ. However, the promoting effect of 4SA-S51A was lower than that of 4SA (Figure 6A). Furthermore, the function of 4SA-S51A in inducing cell proliferation, migration, invasion, and expression of EMT markers was much more attenuated than that of 4SA (Figure 6A, 6B, 6C). These data indicated that TEAD transcription factors played critical roles in mediating the oncogenic function of TAZ in pancreatic cancer cases. To further confirm this, we co-transfected BxPC-3 and AsPC-1 cells with 4SA and TEAD short hairpin RNAs. In addition, as shown in Figure 6D, depletion of TEADs attenuated the regulatory effect of 4SA on the expression of CTGF and EMT markers in these cells.

## DISCUSSION

In the present study, we found that the expression of TAZ was significantly increased in pancreatic cancer tissues as compared to normal pancreas tissues. Further analysis the correlation of TAZ expression with TMA clinicopathologic parameters, we found that the expression of TAZ was positively associated with tumor differentiation. TAZ expression was elevated in pancreatic cancer cell lines as compared to pancreatic ductal epithelial cells. TAZ activation in pancreatic cancer cells promoted pancreatic cancer cells proliferation, migration, invasion and epithelial to mesenchymal transition (EMT). Mechanistically, the aberrant expression and activation of TAZ in pancreatic cancer cells was due to suppression of Merlin expression, which was a positive upstream regulator of the Hippo pathway, and the oncogenic function of TAZ in pancreatic cancer was mediated by the TEAD family transcription factors. Therefore, TAZ functioned as a tumor oncogene and promoted pancreatic cancer EMT and progression. TAZ could be a potential target for designing effective therapeutic strategies.

YAP and TAZ are the predominant transducers of the Hippo pathway, which is a key regulator of tissue growth and cell fate. YAP is reported to be overexpressed in pancreatic cancer cells and to play key roles in tumorigenesis and cancer progression [39-41]. However, the expression and roles of TAZ in pancreatic cancer cases have yet to be studied.

Authors reported that TAZ was overexpressed in a series of cancer cases [7, 42]. In the present study, we found that expression of TAZ was markedly higher in pancreatic tumor specimens and pancreatic cancer cell lines than in normal pancreatic tissue specimens and pancreatic ductal epithelial cells. Further analysis of the correlation of TAZ expression with TMA clinicopathologic parameters demonstrated that the expression was positively associated with tumor differentiation. However, TAZ expression was not correlated with pancreatic cancer TNM stage in our study, which was not consistent with the results of studies of other types of cancer [43]. A larger number of cases than that in the present study is needed for further study.

TAZ expression is reported to be required for cancer cell proliferation, EMT, and metastatic activity [8, 21, 44]. To determine the roles of TAZ in pancreatic cancer biology, we assessed the effect of TAZ on pancreatic cancer proliferation, migration, invasion, and EMT phenotype. We found that elevated expression of TAZ increased BxPC-3 cell colony formation, migration, and invasion and promoted AsPC-1 and BxPC-3 cell EMT via downregulation of E-cadherin and upregulation of vimentin expression. In contrast, depletion of TAZ in FG and PANC-1 cells led to decreased colony formation, migration, and invasion and suppressed the EMT phenotype. These results clearly demonstrated that TAZ functions as an oncogene for pancreatic cancer and promotes pancreatic cancer development and progression.

Our study demonstrated that TAZ expression and activation were elevated in pancreatic cancer cells and promoted cancer development and progression. We further investigated the mechanism of aberrant expression and activation of TAZ in pancreatic cancer cases. Previous studies demonstrated that Merlin was a positive upstream regulator of the Hippo pathway and that expression of Merlin decreased in pancreatic cancer cells [32, 38]. We therefore studied the effect of Merlin on expression and activation of TAZ and found that restored expression of Merlin resulted in decreased expression, nuclear localization, and transcriptional activity of TAZ by increasing phosphorylation of the Hippo pathway kinases MST1 and LATS1 but had a limited effect on 4SA, which has mutations of phosphorylated motifs in the Hippo pathway. These results revealed that Merlin negatively regulated the expression and activation of TAZ via the Hippo pathway and that elevated expression and activation of TAZ in pancreatic cancer cases may result from decreased expression of Merlin.

Authors have reported that TEADs and RUNX2 play important roles in mediation of TAZ's oncogenic effect on tumor development and progression, especially TEADs, which play predominant roles in TAZ-mediated cancer cell proliferation and EMT [19, 36]. By using mutants of TAZ, we found that 4SA-S51A attenuated 4SA-induced pancreatic cancer cell proliferation, migration, invasion, and expression of EMT markers. Furthermore, we found that TEAD short hairpin RNAs suppressed 4SA-induced expression of CTGF and EMT markers. All of these results demonstrated that TEADs play critical roles in mediation of the oncogenic functions of TAZ for pancreatic cancer.

In summary, the present study demonstrated for the first time the mechanism and roles of elevated expression and activation of TAZ in pancreatic cancer development and progression. We present four lines of clinical and experimental evidence supporting this. First, we found that the expression of TAZ increased in pancreatic cancer cells and was positively associated with tumor differentiation. Second, overexpression of TAZ increased pancreatic cancer cell proliferation, migration, invasion, and EMT and subcutaneous tumor growth, whereas depletion of TAZ did the opposite. Third, restored expression of Merlin resulted in decreased expression and activation of TAZ. Fourth, TEADs mediated the oncogenic functions of TAZ in pancreatic cancer cases. With these findings, we identified not only a novel molecular mechanism underlying a novel mechanism of pancreatic cancer development and progression but also a new promising target for early detection and treatment of pancreatic cancer.

## MATERIALS AND METHODS

### Human pancreatic tumor specimens and immunohistochemical analysis

The human pancreatic cancer TMA, which contained 57 primary pancreatic tumor, 10 normal tumor-adjacent pancreatic tissue, and 10 normal pancreatic tissue specimens, was purchased from US Biomax and described previously [34]. Use of the TMA specimens was approved by the relevant institutional review boards. Expression of TAZ was analyzed via staining of the TMA specimens with an anti-TAZ antibody (Cell Signaling Technology). The staining results were scored by two investigators blinded to the clinical data as described previously [45].

### Cell lines

The human pancreatic cancer cell lines PANC-1, MiaPaCa-2, AsPC-1, BxPC-3, and PATU8902 were purchased from the American Type Culture Collection. The pancreatic cancer cell line MDA Panc-28 was a gift from Dr. Paul J. Chiao (The University of Texas MD Anderson Cancer Center). The human pancreatic cancer cell line FG was obtained from Michael P. Vezeridis (The Warren Alpert Medical School of Brown University) [46]. The human metastatic pancreatic cancer cell line Colo357 and its fast-growing liver-metastatic variant in nude mice, L3.7, were described previously [47]. All of these cell lines were maintained in plastic flasks as adherent monolayers in Eagle's minimal essential medium supplemented with 10% fetal bovine serum, sodium pyruvate, nonessential amino acids, L-glutamine, and a vitamin solution (Flow Laboratories). Immortalized human pancreatic ductal epithelial cells (provided by Dr. M.S. Tsao, Ontario Cancer Institute) were maintained in a keratinocyte serum-free medium supplemented with epidermal growth factor and bovine pituitary extract (Invitrogen). The cell lines obtained directly from the American Type Culture Collection were subjected to cell-line characterization or authentication via short tandem repeat profiling and passaged in our laboratory for fewer than 6 months after receipt.

### Plasmids and small interfering RNAs

The plasmids Flag-tagged TAZ (Flag-TAZ), Flag-TAZ-4SA, and Flag-TAZ-4SA-S51A were gifts from Dr. Guan Kunliang (UCLA) and were described previously [19, 36, 37]. Full-length TAZ and its mutants TAZ-4SA and TAZ-4SA-S51A were cloned into pcDNA3.0-HA as *Hin*dIII-*Eco*RI fragments (HA-TAZ, HA-4SA, and HA-4SA-S51A). Full-length TAZ was also cloned into the retroviral expression vector pBABEpuro as a *Bam*HI-*Eco*RI fragment (pBABE-TAZ). PcDNA3.0-Flag-Merlin (pMerlin), the full-length Merlin isoform I, was obtained from Addgene. Retroviruses were produced by transfecting packaging cells (HEK293) with a three-plasmid system consisting of an empty pBABEpuro vector or pBABE-TAZ, the packaging plasmid pUMVC, and the envelope plasmid pCMV-VSV-G. pUMVC and pCMV-VSV-G were obtained from Addgene. Retroviruses were frozen at −20°C or −80°C for long-term storage. Each amplified DNA fragment was verified by sequencing the inserts and flanking regions of the plasmids. Short hairpin RNAs against human TAZ and TEADs were described previously [19, 21]. Sh-TAZ-1 and Sh-TAZ-2 were subcloned into a pLKO.1 puro lentiviral plasmid and verified via direct sequencing. Retroviruses were produced by transfecting packaging cells with pCMV-dR8.2 and pCMV-VSVG, which were obtained from Addgene.

### Gene transfection

For retroviral transduction, pancreatic cancer cells were plated into a six-well plate (∼50% confluent). Twenty-four hours later, the cells were infected with a mixture of retroviruses and hexadimethrine bromide (Polybrene; 5 μg/ml), and stable populations of the cells were obtained via selection with 2 μg/ml puromycin. For transient transfection, cells were transfected with plasmids 48 h before performance of functional assays using Lipofectamine 2000 CD (Invitrogen) [48-50]. Pancreatic cancer cells treated with the transfection reagents alone were included as mock controls.

### Western blot analysis

Total cell lysates and cytoplasmic and nuclear protein fractions were extracted from cell cultures as described previously [51]. Standard Western blot analysis of protein expression was carried out using primary anti-HA (rabbit; Cell Signaling Technology), anti-TAZ (rabbit; Cell Signaling Technology), anti-YAP (rabbit; Cell Signaling Technology), anti-E-cadherin (mouse; BD Biosciences), anti-vimentin (rabbit; Cell Signaling Technology), anti-Merlin (rabbit; Santa Cruz Biotechnology), anti-LATS1 (rabbit; Cell Signaling Technology), anti-MST1 (rabbit; Cell Signaling Technology), anti-phosphorylated LATS1 (Thr1079, rabbit; Cell Signaling Technology), anti-phosphorylated MST1 (Thr183)/MST2 (Thr180, rabbit; Cell Signaling Technology), anti-TEAD (rabbit; Cell Signaling Technology), and anti-CTGF (mouse; Santa Cruz Biotechnology) antibodies. Equal protein-sample loading was monitored using an anti- glyceraldehyde-3-phosphate dehydrogenase (GAPDH) antibody for total cell protein lysates (rabbit; Santa Cruz Biotechnology), an anti-α-tubulin antibody for cytoplasmic fractions (mouse; Oncogene), and an anti-lamin B1 antibody for nuclear fractions (goat; Santa Cruz Biotechnology).

### Colony formation assay

Two hundred cells from each group as indicated were plated in 24-well plates and allowed to grow for 14 days in a culture medium, which was changed twice a week. Cells were then fixed with 4% paraformaldehyde and stained with a 0.1% crystal violet solution for 10 min. Colonies (>20 cells) were counted using an inverted microscope at 40× magnification. All experiments were performed in triplicate and repeated twice [51].

### Animal experiments

Female pathogen-free athymic nude mice were purchased from the National Cancer Institute. The animals were maintained in facilities approved by the Association for Assessment and Accreditation of Laboratory Animal Care International in accordance with the current regulations and standards of the U.S. Department of Agriculture and Department of Health and Human Services. To analyze the growth of pancreatic cancer cells, 1 × 10^6^ cells in 0.1 ml of Hank's balanced salt solution were injected subcutaneously into the right scapular regions of mice. Tumor-bearing mice were killed when they became moribund or on day 35 after inoculation, and their tumors were removed and weighed.

### Cell scratch-wound assay

A cell scratch-wound assay was performed to examine the capacity for pancreatic cancer cells to migrate. Expression of TAZ was altered in BxPC-3 and FG cells by transfecting them with HA-TAZ, Sh-TAZ-1, or control vectors. After the cells grew to 90-95% confluence in six-well plates, the cell surface was wounded by scratching the surface of the plates with a 10-μl pipette tip. Cells were then washed with serum-free Dulbecco's modified Eagle's medium and photographed at 0 and 16 h. *In vitro* wound filling was assessed via measurement of the cell-free areas in multiple fields using a service provided by Wimasis that enables users to upload their images online and have them analyzed and the results uploaded to the users’ servers [52].

### Tumor cell invasion/migration assay

BxPC-3 and FG cells were transfected for 6 h with different reagents respective to different groups (control vector and HA-TAZ or control and Sh-TAZ-1), and AsPC-1 cells were transfected for 6 h with a control, HA-TAZ, HA-4SA, or HA-4SA-S51A. Cells in each group were trypsinized, and 2 × 10^4^ to 5 × 10^4^ cells in a 300-μl volume of serum-free medium in each group were placed in the upper parts of modified Boyden chambers with a Matrigel-coated or uncoated membrane (Millipore). For all cell lines, 500 μl of Dulbecco's modified Eagle's medium with 10% fetal bovine serum was used as a chemoattractant and added to the lower chamber. After 24 h of incubation, invading and/or migrating cells were fixed, stained, and counted under a microscope in five randomly selected fields at a magnification of 200× [53, 54].

### Immunofluorescent staining

Pancreatic cancer cells were seeded on chamber slides overnight. Cells in different groups were transfected with specific plasmids for 48 h, fixed with 4% paraformaldehyde, permeabilized in 0.1% Triton X-100 in phosphate-buffered saline, and sequentially blocked with 3% bovine serum albumin for 30 min. Following overnight incubation with primary antibodies against E-cadherin, the cells were further incubated with appropriate secondary antibodies and subjected to nuclear staining.

### Dual luciferase assay

Pancreatic cancer cells were co-transfected with the 8×GTIIC-luciferase reporter, which included a synthetic TEAD luciferase reporter and was designed for use in examining YAP/TAZ transcriptional activity, pMerlin, HA-TAZ, HA-4SA, or a control vector. The luciferase activity was normalized via co-transfection of those reporters with a β-actin/Renilla luciferase reporter containing a full-length Renilla luciferase gene [55]. The luciferase activity in the cells was quantified using a dual luciferase assay system (Promega) 24 h after transfection.

### Statistical analysis

The two-tailed *X*^2^ test or Fisher exact test was used to determine the significance of differences among the covariates. The significance of the *in vitro* data was determined using the Student *t*-test (two-tailed) or one-way analysis of variance. *P* values less than 0.05 were considered statistically significant. The SPSS software program (version 13.0; IBM Corporation) was used for statistical analysis.
